# Impact of Sn/F Pre-Treatments on the Durability of Protective Coatings against Dentine Erosion/Abrasion

**DOI:** 10.1371/journal.pone.0123889

**Published:** 2015-06-15

**Authors:** Carolina Ganss, Adrian Lussi, Anne Peutzfeldt, Nader Naguib Attia, Nadine Schlueter

**Affiliations:** 1 Department of Conservative and Preventive Dentistry, Dental Clinic of the Justus-Liebig-University Giessen, Giessen, Germany; 2 Department of Preventive, Restorative and Pediatric Dentistry, School of Dental Medicine, University of Bern, Bern, Switzerland; North Carolina A&T State University, UNITED STATES

## Abstract

For preventing erosive wear in dentine, coating with adhesives has been suggested as an alternative to fluoridation. However, clinical studies have revealed limited efficacy. As there is first evidence that Sn^2+^ increases bond strength of the adhesive Clearfil SE (Kuraray), the aim of the present study was to investigate whether pre-treatment with different Sn^2+^/F^−^ solutions improves the durability of Clearfil SE coatings. Dentine samples (eight groups, n=16/group) were freed of smear layer (0.5% citric acid, 10 s), treated (15 s) either with no solution (control), aminefluoride (AmF, 500 ppm F^−^, pH 4.5), SnCl_2_ (800/1600 ppm Sn^2+^; pH 1.5), SnCl_2_/AmF (500 ppm F^−^, 800 ppm Sn^2+^, pH 1.5/3.0/4.5), or Elmex Erosion Protection Rinse (EP, 500 ppm F^−^, 800 ppm Sn^2+^, pH 4.5; GABA International), then rinsed with water (15 s) and individually covered with Clearfil SE. Subsequently the specimens were subjected to an erosion/abrasion protocol consisting of 1320 cycles of immersion in 0.5% citric acid (5°C/55°C; 2 min) and automated brushing (15 s, 200 g, NaF-toothpaste, RDA 80). As the coatings proved stable up to 1320 cycles, 60 modified cycles (brushing time 30 min/cycle) were added. Wear was measured profilometrically. After SnCl_2_/AmF, pH 4.5 or EP pre-treatment all except one coating survived. In the other groups, almost all coatings were lost and there was no significant difference to the control group. Pre-treatment with a Sn^2+^/F^−^ solution at pH 4.5 seems able to improve the durability of adhesive coatings, rendering these an attractive option in preventing erosive wear in dentine.

## Introduction

Fluoride and stannous ions have been successfully used as preventive agents against enamel erosion. Reductions in tissue loss in the order of 64–91% have been reported after application of rinses [1] and of 55–67% after application of toothpaste slurries [2]. In dentine, however, such formulations are much less effective [3–5] highlighting the need for seeking new approaches for patients with advanced erosive wear into dentine.

An alternative to topical Sn^2+^/F^-^ application is coating dentine lesions with sealants or adhesives [6], and in vitro studies have indeed demonstrated promising protective effects [7–9]. Thus, in a study comparing an adhesive and two resin-based desensitisers, dentine samples were either left untreated or covered with Optibond FL (Kerr, Orange, CA, USA), Seal&Protect (Dentsply DeTrey, Konstanz, Germany), or an experimental version of the latter. Even after eight days of a recurrent cycling procedure consisting of 3 hours immersion in HCl (pH 3.0) and 600 brushing strokes, almost complete protection was observed [9]. Another study found coatings with Seal&Protect to be more effective than 5 min pre-treatment with a 0.05% sodium fluoride solution when subjected to a wear regime for up to 50 cycles of immersion in 0.3% citric acid for 5 min followed by 100 brushing strokes [7]. Furthermore, in situ, coatings with Seal&Protect or Optibond Solo were found to survive for 20 days an erosion protocol consisting of daily immersion in 0.05 M citric acid for 24 min [10].

In contrast, two clinical studies using a split mouth design have shown much less promising results. In one study [6] 19 patients with tooth wear of unspecified aetiology were included and had one tooth sealed (Seal&Protect) whereas the corresponding tooth was left untreated. For the first 6 months, the wear rate of the non-treated control teeth was slightly higher than that of the sealed teeth, whereas after 6 months, there was no significant difference between sealed teeth and control teeth. Based on these results, another study [11], using a similar study design, investigated whether a fissure sealant (Helioseal Clear Chroma, Ivoclar/Vivadent, Schaan, Liechtenstein) used together with a self-etching adhesive (AdheSE, Ivoclar/Vivadent, Schaan, Liechtenstein) would provide longer protection than Seal&Protect. Though the tissue loss values were significantly lower in the sealed teeth for up to nine months, most sealants were lost already after 6 months in service. To sum up, these results indicate that adhesive and sealant coatings have the potential to prevent erosive tooth wear, but also that their durability is limited and needs to be improved especially considering that this is an in-office procedure.

In this context, an interesting finding was that the bond strength of Clearfil SE, which is a two-step self-etch adhesive system containing 10-methacryloyloxydecyl dihydrogen phosphate (MDP), was distinctly higher to eroded dentine samples, which had undergone repetitive cycles of de- and remineralisation and applications of a AmF/NaF/SnCl_2_ solution (500 ppm F^-^, 800 ppm Sn^2+^) than to samples, which had been treated with a NaF solution (500 ppm F^-^) or to control samples [12]. The repetitive application of the AmF/NaF/SnCl_2_ used in this experiment, however, is not feasible as a pre-treatment prior to surface coating. Therefore, another experiment investigated the effect of a single pre-treatment with a 35% SnCl_2_ solution. The study revealed promising results, but the positive effect was limited [13]. It was speculated that this was either due to the high concentration, the high acidity, or the absence of fluoride, and thus several questions remained open: is it the Sn-ion per se which causes the beneficial effect, or is the presence of fluoride essential? What is the optimal concentration and does the pH play a role? Therefore, the present study aims to investigate whether a single pre-treatment with a low concentrated solution of SnCl_2_ or AmF, or combinations hereof and at different pH, increases the durability of protective Clearfil SE coatings under erosive/abrasive conditions.

## Materials and Methods

The principal experimental design and procedures are performed according our standardised protocols and are described earlier [4]. The use of human samples was approved by the local Independent Ethics Committee of the Justus-Liebig-University Giessen (143/09). Previously impacted human third molars removed for other reasons were used. Oral informed consent from the donors was approved sufficient by this Ethics Committee as was waiver of written documentation of consent.

### Preparation of dentine specimens

After extraction, the teeth were stored in saturated thymol solution (Thymol, Fluka Chemie AG, Buchs, Switzerland) until use. After removing the enamel from the smooth surfaces, dentine slices were prepared (Exakt Abrasive Cutting System and Exakt Microgrinder, Exakt-Apparatebau, Norderstedt, Germany; silicon carbide polishing discs, 15 and 3 μm; Leco, Michigan, USA) and the resulting outer dentine surface was freed of smear layer (0.5% citric acid, 10 s). Samples were mounted with resin onto sample holders (Technovit 7230 VLC, Kulzer-Exakt, Wehrheim, Germany) suitable for an automated brushing machine (SD Mechatronik GmbH, Feldkirchen-Westerham, Germany).

The samples were randomly assigned to eight groups (n = 16/group) and gently brushed with a disposable brush (Kuraray Dental, Chiyoda, TKY, Japan) for 15 s with one of the following solutions: AmF (500 ppm F^-^; pH 4.5), SnCl_2_ (800 or 1600 ppm Sn^2+^; pH 1.5 each), SnCl_2_/AmF (500 ppm F^-^, 800 ppm Sn^2+^; adjusted to pH 1.5 or 3.0 or 4.5 with 0.1 M HCl), or Elmex Erosion Protection Rinse (EP, 250 ppm F^-^ as AmF, 250 ppm F^-^ as NaF, 800 ppm Sn^2+^; pH 4.5, CP GABA GmbH, Hamburg, Germany). After application of the solutions, samples were rinsed with tap water for 15 s with a dental syringe. Samples of the control group received no pre-treatment.

Subsequently, the samples were gently air dried and individually coated with Clearfil SE (Table 1) according to the manufacturer’s instructions for use and light cured for 10 s (Elipar FreeLight 2, 3M ESPE, Neuss, Germany). As the Clearfil SE might potentially have spread beyond the sample surface, coated samples were carefully separated from the surrounding mounting resin with a diamond bur (fine grain diamond 88 89 010; Komet Dental Gebr. Brasseler, Lemgo, Germany). Finally, samples were checked for any imperfection under a microscope (SMZ-2T, Nikon, Tokyo, Japan). In case of damage, samples were discarded.

**Table 1:** Composition and mode of application of Clearfil SE according to the manufacturer.

Composition Primer	Composition Adhesive	Mode of application
MDP (10-methacryloyloxydecyl dihydrogen phosphate), HEMA (2-hydroxyethyl methacrylate), hydrophilic dimethacrylate, dl-camphorquinone, N,N-diethanol-p-toluidine, water	MDP (10-methacryloyloxydecyl dihydrogen phosphate, Bis-GMA (bis-phenol A diglycidylmethacrylate), HEMA (2-hydroxyethyl methacrylate), hydrophobic dimethacrylate,dl-camphorquinone, N,N-diethanol-p-toluidine silanated colloidal silica	Primer: 20 s application; gentle air blowing for a few seconds
		Adhesive: application, gentle air blowing for a few seconds, 20 s light curing

### Experimental procedure

The acid impacts were produced by immersing the samples in 0.5% citric acid (citric acid monohydrate; Merck, Darmstadt, Germany; natural pH 2.5 alternating at 5°C and 55°C) for 2 min in a water bath (Model 1083, GFL mbH, Burgwedel, Germany). The samples were rinsed with tap water for 30 s and then inserted into an automated brushing machine equipped with standardised brushes (ADA reference brush soft). The brush moved in a "zig-zag" pattern (150 oscillations/min, linear travel path 6 mm, travel velocity 60 mm/s). Brushing was performed for 15 s under a load of 200 g and with a NaF-toothpaste slurry (1450 ppm F^-^, RDA 80, 1 part toothpaste: 3 parts distilled water; Dentagard Original, Colgate-Palmolive, Hamburg, Germany) for a total of 1320 cycles. One cycle consisted of one acid impact and one brushing procedure. As the coatings proved to be stable up to 1320 impacts, the samples were subsequently subjected to 60 modified cycles in which the toothbrushing was increased from 15 s to 30 min.

All solutions and slurries were freshly prepared at the beginning of each experimental day.

### Profilometry

The sample holders were equipped with stripes of stainless steel serving as reference areas and allowing re-identification of the traced area for monitoring.

Wear of the coatings was quantified with an optical device (MicroProf, Fries Research & Technology GmbH, Bergisch-Gladbach, Germany); measurements were performed prior to coating (baseline), immediately after coating and then after 60, 120, 300, 480, 840, and 1320 cycles as well as after 20 and 60 modified cycles. Three traces were made at intervals of 0.2 mm for a total length of 4 mm (200 pixel, 32 hertz, sensor HO). For standardised moisture control, a drop of distilled water was applied for 30 s and removed with absorbent paper prior to each tracing. Traces were analysed with special software (Mark III, Fries Research & Technology GmbH Bergisch-Gladbach, Germany). Two parallel regression lines were constructed for each trace. One regression line was drawn on the reference area, and one regression line was drawn on the experimental area; both lines were 0.5 mm in length. The vertical distance between the regression lines was defined as the step height between reference area and experimental area (μm; mean of three traces). Initial coating thickness, wear of coating and tissue loss were calculated as the difference between the value of the baseline measurement and the value measured at the respective time point. The twentyfold measurement of a sample, which was removed and re-positioned prior to each new measurement, showed a step height of 477 μm and a standard deviation of ±4 μm.

### Statistics

For the statistical procedures, IBM SPSS statistics, version 22 (SPSS GmbH, Munich, Germany) was used. There was no significant deviation from the Gaussian distribution (Kolmogorov-Smirnov-test). Comparisons between groups, i.e. between the various pre-treatments, were performed with ANOVA followed by Tamhane’s post hoc tests as there was a significant deviation for the homogeneity of variance (Levene’s test). The level of significance was set at .05. Comparisons within groups, i.e. between the various numbers of erosive/abrasive cycles, were performed with paired t-tests (level of significance after Bonferroni-adjustment: 0.005).

## Results

**Fig 1:**
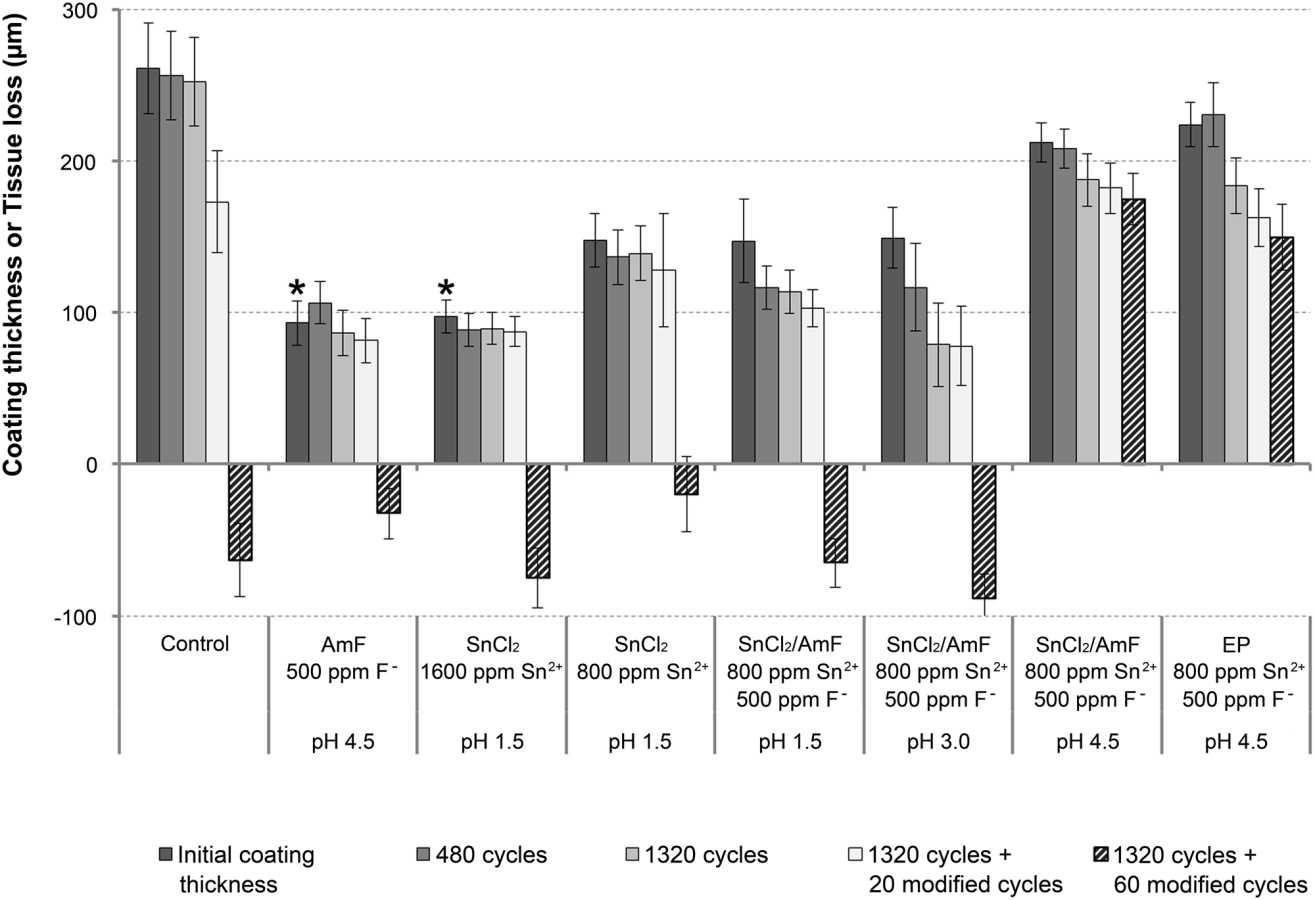
Thickness of the Clearfil SE coatings (positive values) and tissue loss (negative values) after the various pre-treatments of smear layer-freed dentine (mean±SE). * indicates significant reduction of initial coating thickness compared to the control group.

Coating thickness and tissue loss values are shown in Fig 1. Pre-treatments led to numerically thinner initial coatings compared to control, which reached significance for treatments with AmF or 1600 ppm SnCl_2_. Within group effects were minor until 1320 + 20 modified cycles, but after 1320 + 60 modified cycles significant dentine wear occurred in all groups except in those with pre-treatments of either the SnCl_2_/AmF solution at pH 4.5 or the EP. In these two groups, coatings were retained throughout the experiment (except for one sample in the EP group); in all other groups the majority of the coatings had been lost (Table 2) as indicated by negative profilometric values (Fig 1). There were no significant differences between group effects at 1320 + 60 modified cycles except for the SnCl_2_/AmF solution at pH 4.5 and EP, which exhibited positive values compared to negative values in all other groups. These two solutions were of similar effectiveness (n.s.). For the sake of clarity, data for 60, 120, 300, and 840 cycles are not shown because they did not provide any additional information.

**Table 2:** Number of completely lost coatings after 480, 1320, as well as 1320+20 or 1320+60 modified cycles indicated by tissue loss (negative profilometric values).

Cycles	Control	AmF	SnCl_2_ 800 ppm Sn^2+^	SnCl_2_ 1600 ppm Sn^2+^	SnCl_2_/AmF pH 1.5	SnCl_2_/AmF pH 3.0	SnCl_2_/AmF pH 4.5	EP
480	0 (16)	0 (16)	0 (16)	0 (16)	0 (15)	2 (16)	0 (14)	0 (16)
1320	0 (16)	0 (16)	0 (16)	0 (16)	0 (15)	3 (16)	0 (14)	0 (16)
1320+20	2 (16)	1 (16)	2 (16)	0 (16)	0 (15)	5 (16)	0 (14)	0 (16)
1320+60	11 (16)	10 (16)	8 (16)	14 (16)	13 (15)	16 (16)	0 (14)	1 (15)

Total number of samples is given in brackets.

## Discussion

To date, there is no established experimental protocol for studies investigating the effect of coating dental hard tissues against erosive/abrasive impacts. We therefore designed a procedure including thermal, mechanical, and acidic impacts as they might occur in the oral cavity and used dentine surfaces mimicking the in vivo histology of erosion (for the histological structure of eroded dentine see [14]). The latter is substantially different from bur-treated, smear layer-covered dentine for which adhesives are designed. Indeed, a study of long-term bond strength to dentine found much lower bond strength to eroded dentine than to smear layer-covered dentine [15]. Consequently, we chose to remove the smear layer by brief immersion in citric acid. Preliminary experiments using scanning electron microscopy combined with energy dispersive X-ray spectroscopy verified that this acid treatment selectively removed the smear layer without demineralising the underlying dentine to such an extent that collagen was exposed (see S1 and S2 Figs, and S1 Table).

The erosion and abrasion protocol followed previous experiments [4], except that thermocycling was now included to mimic more closely real life conditions. The experiment was run continuously for approximately 4 months which means that the coatings were subject to ageing. Except for the solution with a Sn concentration of 1600 ppm, the concentration of Sn and F in the experimental solutions was identical to that used in the previous study [12]. AmF was chosen because of its ability to keep SnCl_2_ in solution even at higher pH.

Overall, coatings with Clearfil SE were stable over more than 1300 cycles indicating that this material is capable of providing durable protection under in vitro conditions. When the protective effect was lost, this was not due to wearing away of the coating material, but to bond failure. This indicates that improving the adhesion is the key to improving the longevity of these coatings.

Clearfil SE is an established adhesive which has been extensively studied [16, 17], and its mode of action is quite well understood. The acidic phosphate monomer (MDP) in a first step adsorbs to hydroxyapatite (HAp) simultaneously causing some mineral dissolution. It also binds covalently through condensation with PO_4_^3-^ of HAp. A further mechanism contributing to adhesion is self-assembling of two MDP molecules linked together by a Ca-ion forming nano-layers [18]. The latter has not only been demonstrated for the interaction with pure HAp, but occurs also in natural dentine [19].

So far, the mode of action of the active agents applied in this study remains unclear. The pre-treatment with AmF had no effect on coating durability which means on one hand that it is not the fluoride ion as such which improves retention but on the other hand that fluoride applications, as often recommended in cases of erosive loss and/or hypersensitivity, do not determine the success of the coatings. SnCl_2_ was also not influential. Considering that etching dentine with phosphoric acid for 15 s has been shown to significantly decrease the bond strength of Clearfil SE [20], a possible explanation for the lack of influence of SnCl_2_ could be the relatively low pH causing undesired etching. Adding fluoride to SnCl_2_ solutions had no impact at pH values of 1.5 and 3.0. This indicates that it is probably the pH of the Sn^2+^-solutions rather than the presence of F^-^ which is crucial. All these solutions decreased the thickness of the coatings, at least numerically, without affecting the durability, and this could be an advantage for treating occluding areas of teeth.

When the pH was raised to 4.5, however, the SnCl_2_/AmF pre-treatment increased the durability of the coatings markedly as did pre-treatment with the EP mouthrinse.

In principle, there are two explanatory approaches to the positive effect of the SnCl_2_/AmF, pH 4.5 solution. One regards the composition of the mineral phase of dentine. Sn^2+^ is readily adsorbed to HAp; its ionic radius (0.71 Å) differs only slightly from that of Ca^2+^ (0.99 Å), and it can substitute Ca^2+^ in the HAp crystal lattice [21]. Thus, it can be speculated that the ionic binding of the phosphate group of MDP to Sn^2+^-doped HAp is stronger and that MDP-Sn salts are more stable. This is corroborated by the finding that pre-treatment of enamel with 35% SnCl_2_ increased the bond strength of Clearfil SE distinctly without generating any retentive etching pattern [22].

The other approach of explanation regards the components of the adhesive. In Clearfil SE, the molecule that contributes the most to adhesion is MDP, but the primer also contains HEMA. HEMA is a small hydrophilic monomer ensuring wetting, thus facilitating the diffusion of the adhesive into the dentine substrate [23]. However, it has been shown that HEMA adversely interacts with MDP. HEMA has been found not to prevent MDP from adsorbing to HAp, but seems to inhibit MDP-Ca salts and the build-up of nano-layers [24]. Also, with respect to reactions with collagen, adverse effects were found, and it was speculated that HEMA and MDP form aggregates via hydrogen and electrostatic bonding, thus compromising the MDP-collagen interaction [25]. In the slightly acidic environment of the primer, Sn-ions could provide a source of charged elements potentially influencing these interactions.

## Conclusions

Coating dentine with the adhesive Clearfil SE prevented tissue loss from acid and abrasion impacts. A single pre-treatment with a SnCl_2_/AmF solution at pH 4.5 or with EP increased the durability of these coatings significantly and offers a promising option for treating advanced erosive wear into dentine. More research is necessary to elucidate the mode of action as well as to investigate the efficacy of such coatings in vivo.

## Supporting Information

S1 Dataset (PDF)

S1 Fig SEM picture of a representative dentine surface after preparation. An amorphous smear layer as well as scoring marks are clearly visible. Original magnification x5000.
(TIF)

S2 Fig SEM picture of a representative dentine surface after removal of the smear layer. Smear layer was removed by immersion in 0.5% citric acid, natural pH 2.5, for 10 s. Open tubules are clearly visible, the peritubular dentin is preserved. Energy Dispersive X-ray Spectroscopy analysis indicates that there was no relevant demineralisation of the resulting dentine surface (see S1 Table). Original magnification x5000.
(TIF)

S1 Table Energy Dispersive X-ray Spectroscopy analysis. C, O, P and Ca on dentine surfaces (n = 10 each; wt%, mean±standard deviation) after preparation (with smear layer) and after treatment with 0.5% citric acid (natural pH 2.5) for 10 s (without smear layer). Samples were dried at ambient air, sputter-coated with gold (JFC-1200 fine coater, Tokyo, Japan; 90 s, 40 mA) and investigated at 2000-fold original magnification (JSM-6510, Jeol, Tokyo, Japan equipped with a X-Flash Detector 410-M, Bruker Nano GmbH, Berlin, Germany; acceleration voltage 15 kV, count rates ~1 kcps). Groups sharing the same superscript letter (columns) are not significantly different (t-test for independent samples)
(DOC)
