# Supplementary material for: Impact of Sn/F Pre-Treatments on the Durability of Protective Coatings against Dentine Erosion/Abrasion
Source: PLoS One. 2015 Jun 15;10(6):e0123889. doi: 10.1371/journal.pone.0123889 (PMC4468142; doi:10.1371/journal.pone.0123889)
Supplement: S1 Dataset — (PDF) [file pone.0123889.s001.pdf]

Treatment	Initial coating thickness	480 cycles	1320 cycles	1320cycles +20 modif. cycles	1320cycles +60 modif. cycles
Control	426,33	409,33	392,00	174,67	-192,33
Control	366,67	363,33	356,33	218,67	-112,00
Control	301,67	288,67	278,00	-20,67	-223,00
Control	441,33	433,33	417,67	-95,67	21,33
Control	283,67	277,67	296,67	399,67	149,33
Control	435,67	432,67	427,67	436,67	-15,67
Control	286,33	273,00	288,00	299,00	-104,00
Control	374,00	372,33	373,33	161,00	-61,00
Control	86,33	82,67	64,67	64,67	-169,00
Control	162,33	151,00	132,00	126,00	-137,67
Control	156,67	150,00	134,67	121,00	-125,00
Control	132,67	153,67	125,67	152,33	17,00
Control	166,00	156,33	179,67	173,67	16,00
Control	184,67	195,00	204,67	191,00	-41,00
Control	224,33	223,33	227,67	230,00	9,67
Control	151,67	141,00	141,00	137,00	-45,67
AmF 500 ppm F; pH 4.5	86,33	84,00	91,00	61,33	0,00
AmF 500 ppm F; pH 4.5	204,67	232,67	222,00	190,67	-102,00
AmF 500 ppm F; pH 4.5	143,67	151,33	137,00	134,67	29,33
AmF 500 ppm F; pH 4.5	181,33	167,67	151,00	156,33	61,33
AmF 500 ppm F; pH 4.5	14,00	26,00	20,67	-1,67	-145,00
AmF 500 ppm F; pH 4.5	108,00	103,67	90,33	90,67	-76,00
AmF 500 ppm F; pH 4.5	64,33	69,67	63,33	55,33	-47,33
AmF 500 ppm F; pH 4.5	187,00	181,00	159,67	156,67	12,00
AmF 500 ppm F; pH 4.5	89,67	94,67	85,33	93,00	22,33
AmF 500 ppm F; pH 4.5	62,33	72,00	53,33	52,67	-26,33

AmF 500 ppm F; pH 4.5	25,33	35,67	14,67	21,00	-38,33
AmF 500 ppm F; pH 4.5	57,33	79,67	62,67	73,33	-40,67
AmF 500 ppm F; pH 4.5	112,33	158,67	132,33	144,67	88,67
AmF 500 ppm F; pH 4.5	39,00	80,67	30,33	28,00	-122,67
AmF 500 ppm F; pH 4.5	51,00	74,00	26,00	14,33	-109,33
AmF 500 ppm F; pH 4.5	64,00	91,00	44,67	33,33	-27,33
SnCl2 800 ppm Sn; pH 1.5	-6,00	22,33	44,33	55,67	-119,00
SnCl2 800 ppm Sn; pH 1.5	258,00	284,67	289,67	283,67	111,00
SnCl2 800 ppm Sn; pH 1.5	169,67	183,67	182,33	170,00	-41,33
SnCl2 800 ppm Sn; pH 1.5	65,33	59,67	64,33	60,00	31,00
SnCl2 800 ppm Sn; pH 1.5	165,67	133,67	134,67	70,67	-55,33
SnCl2 800 ppm Sn; pH 1.5	105,33	61,00	45,33	38,67	71,00
SnCl2 800 ppm Sn; pH 1.5	132,67	116,00	133,00	122,33	42,00
SnCl2 800 ppm Sn; pH 1.5	131,67	112,00	129,00	126,67	-9,00
SnCl2 800 ppm Sn; pH 1.5	85,33	83,00	64,00	408,00	-175,00
SnCl2 800 ppm Sn; pH 1.5	250,00	241,33	241,33	293,00	-60,33
SnCl2 800 ppm Sn; pH 1.5	164,00	144,33	146,00	263,00	29,00
SnCl2 800 ppm Sn; pH 1.5	84,00	73,33	62,67	-37,00	-242,67
SnCl2 800 ppm Sn; pH 1.5	149,33	118,00	133,00	-207,00	-99,33
SnCl2 800 ppm Sn; pH 1.5	195,00	180,00	190,00	86,00	63,00
SnCl2 800 ppm Sn; pH 1.5	195,00	173,00	161,67	47,67	64,00
SnCl2 800 ppm Sn; pH 1.5	217,33	198,67	204,00	267,33	73,33
SnCl2 1600 ppm Sn; pH 1.5	137,33	121,00	119,33	113,00	-42,00
SnCl2 1600 ppm Sn; pH 1.5	19,67	17,33	11,33	9,67	-158,67
SnCl2 1600 ppm Sn; pH 1.5	152,33	141,67	137,33	127,00	-26,00
SnCl2 1600 ppm Sn; pH 1.5	113,67	111,00	106,67	99,00	53,67
SnCl2 1600 ppm Sn; pH 1.5	122,00	93,33	114,67	98,33	-142,67
SnCl2 1600 ppm Sn; pH 1.5	157,67	154,67	153,00	143,33	-102,67

SnCl2 1600 ppm Sn; pH 1.5	98,67	76,00	81,33	83,33	34,67
SnCl2 1600 ppm Sn; pH 1.5	56,33	50,33	48,33	46,67	-22,00
SnCl2 1600 ppm Sn; pH 1.5	46,33	71,33	76,67	120,33	-243,00
SnCl2 1600 ppm Sn; pH 1.5	37,67	28,00	33,00	30,00	-42,33
SnCl2 1600 ppm Sn; pH 1.5	75,67	58,33	57,67	61,33	-81,67
SnCl2 1600 ppm Sn; pH 1.5	79,33	65,33	72,00	59,00	-52,33
SnCl2 1600 ppm Sn; pH 1.5	104,67	91,33	103,00	81,00	-142,00
SnCl2 1600 ppm Sn; pH 1.5	97,00	91,00	83,67	94,67	-53,67
SnCl2 1600 ppm Sn; pH 1.5	93,33	76,00	69,67	80,33	-23,00
SnCl2 1600 ppm Sn; pH 1.5	167,00	170,33	165,33	152,00	-159,00
SnCl2/AmF 800 ppm Sn + 500 ppm F; pH 4.5					
SnCl2/AmF 800 ppm Sn + 500 ppm F; pH 4.5	297,00	250,00	282,67	286,67	270,67
SnCl2/AmF 800 ppm Sn + 500 ppm F; pH 4.5	213,67	169,67	176,33	180,00	167,33
SnCl2/AmF 800 ppm Sn + 500 ppm F; pH 4.5					
SnCl2/AmF 800 ppm Sn + 500 ppm F; pH 4.5	177,67	205,67	122,00	138,00	123,00
SnCl2/AmF 800 ppm Sn + 500 ppm F; pH 4.5	164,00	201,00	110,33	95,00	105,67
SnCl2/AmF 800 ppm Sn + 500 ppm F; pH 4.5	196,00	226,67	148,33	133,67	144,67
SnCl2/AmF 800 ppm Sn + 500 ppm F; pH 4.5	234,67	255,67	153,33	157,67	142,67
SnCl2/AmF 800 ppm Sn + 500 ppm F; pH 4.5	161,00	139,67	134,00	110,67	95,33
SnCl2/AmF 800 ppm Sn + 500 ppm F; pH 4.5	235,00	218,33	206,33	178,67	166,67
SnCl2/AmF 800 ppm Sn + 500 ppm F; pH 4.5	215,00	191,67	192,00	182,00	177,00
SnCl2/AmF 800 ppm Sn + 500 ppm F; pH 4.5	159,00	131,33	119,67	144,67	118,00

SnCl2/AmF 800 ppm Sn + 500 ppm F; pH 4.5	287,33	282,00	302,67	279,00	276,00
SnCl2/AmF 800 ppm Sn + 500 ppm F; pH 4.5	272,33	273,33	289,67	278,67	277,33
SnCl2/AmF 800 ppm Sn + 500 ppm F; pH 4.5	201,00	214,33	223,67	219,67	215,67
SnCl2/AmF 800 ppm Sn + 500 ppm F; pH 4.5	156,33	154,67	166,00	167,00	169,00
SnCl2/AmF 800 ppm Sn + 500 ppm F; pH 1.5	117,33	120,00	122,67	117,00	-81,67
SnCl2/AmF 800 ppm Sn + 500 ppm F; pH 1.5	114,67	118,00	118,67	106,33	-108,33
SnCl2/AmF 800 ppm Sn + 500 ppm F; pH 1.5	40,67	34,67	55,00	52,67	-74,67
SnCl2/AmF 800 ppm Sn + 500 ppm F; pH 1.5	33,00	31,33	32,67	34,33	-100,33
SnCl2/AmF 800 ppm Sn + 500 ppm F; pH 1.5	156,33	154,00	145,67	141,67	-70,33
SnCl2/AmF 800 ppm Sn + 500 ppm F; pH 1.5	113,33	111,00	108,33	104,33	-52,00
SnCl2/AmF 800 ppm Sn + 500 ppm F; pH 1.5	66,00	61,00	53,33	47,33	-89,00
SnCl2/AmF 800 ppm Sn + 500 ppm F; pH 1.5	75,00	73,33	80,33	75,33	-104,33
SnCl2/AmF 800 ppm Sn + 500 ppm F; pH 1.5	151,00	200,67	213,33	166,33	-79,00
SnCl2/AmF 800 ppm Sn + 500 ppm F; pH 1.5	118,33	168,67	167,00	161,00	69,33
SnCl2/AmF 800 ppm Sn + 500 ppm F; pH 1.5	105,33	154,67	132,00	117,33	-102,67
SnCl2/AmF 800 ppm Sn + 500 ppm F; pH 1.5	90,67	143,33	157,00	123,00	-4,33
SnCl2/AmF 800 ppm Sn + 500 ppm F; pH 1.5					
SnCl2/AmF 800 ppm Sn + 500 ppm F; pH 1.5	331,00	174,67	169,00	136,33	-47,67

SnCl2/AmF 800 ppm Sn + 500 ppm F; pH 1.5	369,67	154,67	136,33	146,67	41,33
SnCl2/AmF 800 ppm Sn + 500 ppm F; pH 1.5	327,00	42,67	14,00	15,67	-173,33
SnCl2/AmF 800 ppm Sn + 500 ppm F; pH 3.0	216,67	122,33	134,00	124,67	-111,00
SnCl2/AmF 800 ppm Sn + 500 ppm F; pH 3.0	112,33	1,67	-38,00	-18,67	-79,67
SnCl2/AmF 800 ppm Sn + 500 ppm F; pH 3.0	66,67	-48,33	-84,33	-52,67	-4,33
SnCl2/AmF 800 ppm Sn + 500 ppm F; pH 3.0	138,33	-22,33	14,00	-18,33	-144,00
SnCl2/AmF 800 ppm Sn + 500 ppm F; pH 3.0	270,00	241,00	226,00	239,33	-4,00
SnCl2/AmF 800 ppm Sn + 500 ppm F; pH 3.0	61,33	7,67	2,67	-7,00	-150,67
SnCl2/AmF 800 ppm Sn + 500 ppm F; pH 3.0	102,33	48,67	34,00	41,33	-32,33
SnCl2/AmF 800 ppm Sn + 500 ppm F; pH 3.0	164,00	308,33	163,67	234,33	-28,67
SnCl2/AmF 800 ppm Sn + 500 ppm F; pH 3.0	126,33	120,00	109,00	87,67	-176,00
SnCl2/AmF 800 ppm Sn + 500 ppm F; pH 3.0	79,33	53,00	49,33	56,67	-48,00
SnCl2/AmF 800 ppm Sn + 500 ppm F; pH 3.0	371,33	372,67	361,67	299,33	-46,00
SnCl2/AmF 800 ppm Sn + 500 ppm F; pH 3.0	146,00	123,33	113,33	114,33	-79,67
SnCl2/AmF 800 ppm Sn + 500 ppm F; pH 3.0	90,67	99,33	-19,33	-25,67	-68,33
SnCl2/AmF 800 ppm Sn + 500 ppm F; pH 3.0	142,67	144,67	43,67	36,00	-125,33
SnCl2/AmF 800 ppm Sn + 500 ppm F; pH 3.0	134,00	135,00	48,00	18,33	-230,67
SnCl2/AmF 800 ppm Sn + 500 ppm F; pH 3.0	164,33	159,67	105,33	116,67	-86,33
EP 800 ppm Sn + 500 ppm F; pH 4.5	129,67	126,67	92,00	58,00	71,67

EP 800 ppm Sn + 500 ppm F; pH 4.5	145,00	113,33	67,33	58,00	
EP 800 ppm Sn + 500 ppm F; pH 4.5	224,33	197,33	176,33	158,33	144,33
EP 800 ppm Sn + 500 ppm F; pH 4.5	251,00	221,33	188,67	197,00	168,67
EP 800 ppm Sn + 500 ppm F; pH 4.5	268,33	242,00	242,33	235,00	237,67
EP 800 ppm Sn + 500 ppm F; pH 4.5	300,67	268,33	260,00	267,00	272,33
EP 800 ppm Sn + 500 ppm F; pH 4.5	247,00	219,33	207,33	190,67	188,33
EP 800 ppm Sn + 500 ppm F; pH 4.5	167,00	138,67	126,33	128,00	125,33
EP 800 ppm Sn + 500 ppm F; pH 4.5	235,33	386,33	243,67	234,00	245,33
EP 800 ppm Sn + 500 ppm F; pH 4.5	198,33	275,00	242,67	162,67	243,00
EP 800 ppm Sn + 500 ppm F; pH 4.5	151,33	227,33	64,00	30,33	70,33
EP 800 ppm Sn + 500 ppm F; pH 4.5	324,67	420,00	319,00	276,33	203,67
EP 800 ppm Sn + 500 ppm F; pH 4.5	187,33	170,67	176,00	204,33	143,00
EP 800 ppm Sn + 500 ppm F; pH 4.5	253,00	264,67	205,67	175,33	105,67
EP 800 ppm Sn + 500 ppm F; pH 4.5	209,33	182,00	108,67	74,33	-15,00
EP 800 ppm Sn + 500 ppm F; pH 4.5	294,00	239,33	220,00	157,67	43,00

Missing values are lost dentine samples during brushing procedures
