# Supplementary figures and images for: Impact of Sn/F Pre-Treatments on the Durability of Protective Coatings against Dentine Erosion/Abrasion
Source: PLoS One. 2015 Jun 15;10(6):e0123889. doi: 10.1371/journal.pone.0123889 (PMC4468142; doi:10.1371/journal.pone.0123889)

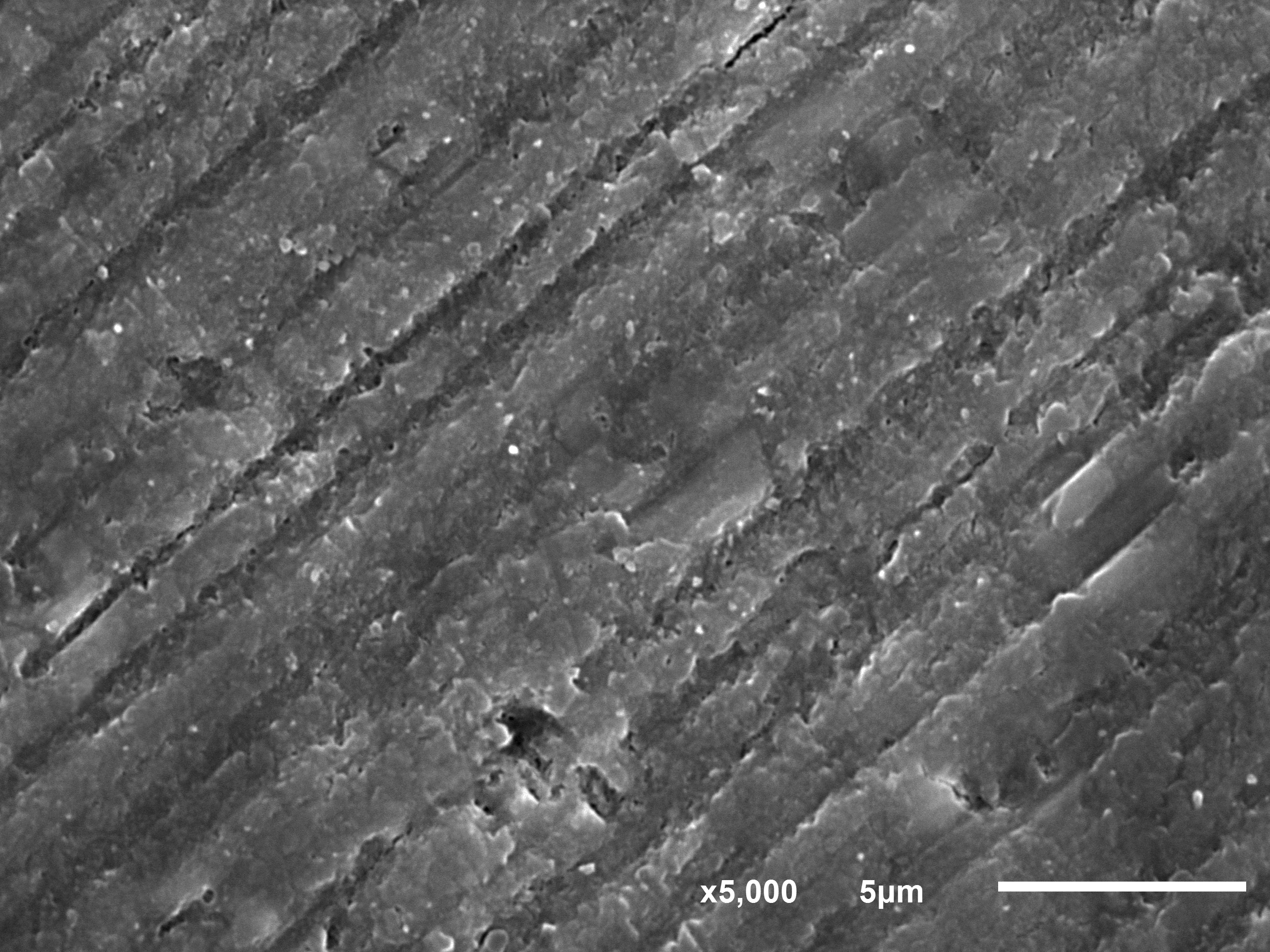

Supplement: S1 Fig — An amorphous smear layer as well as scoring marks are clearly visible. Original magnification x5000. (TIF) [file pone.0123889.s002.tif]

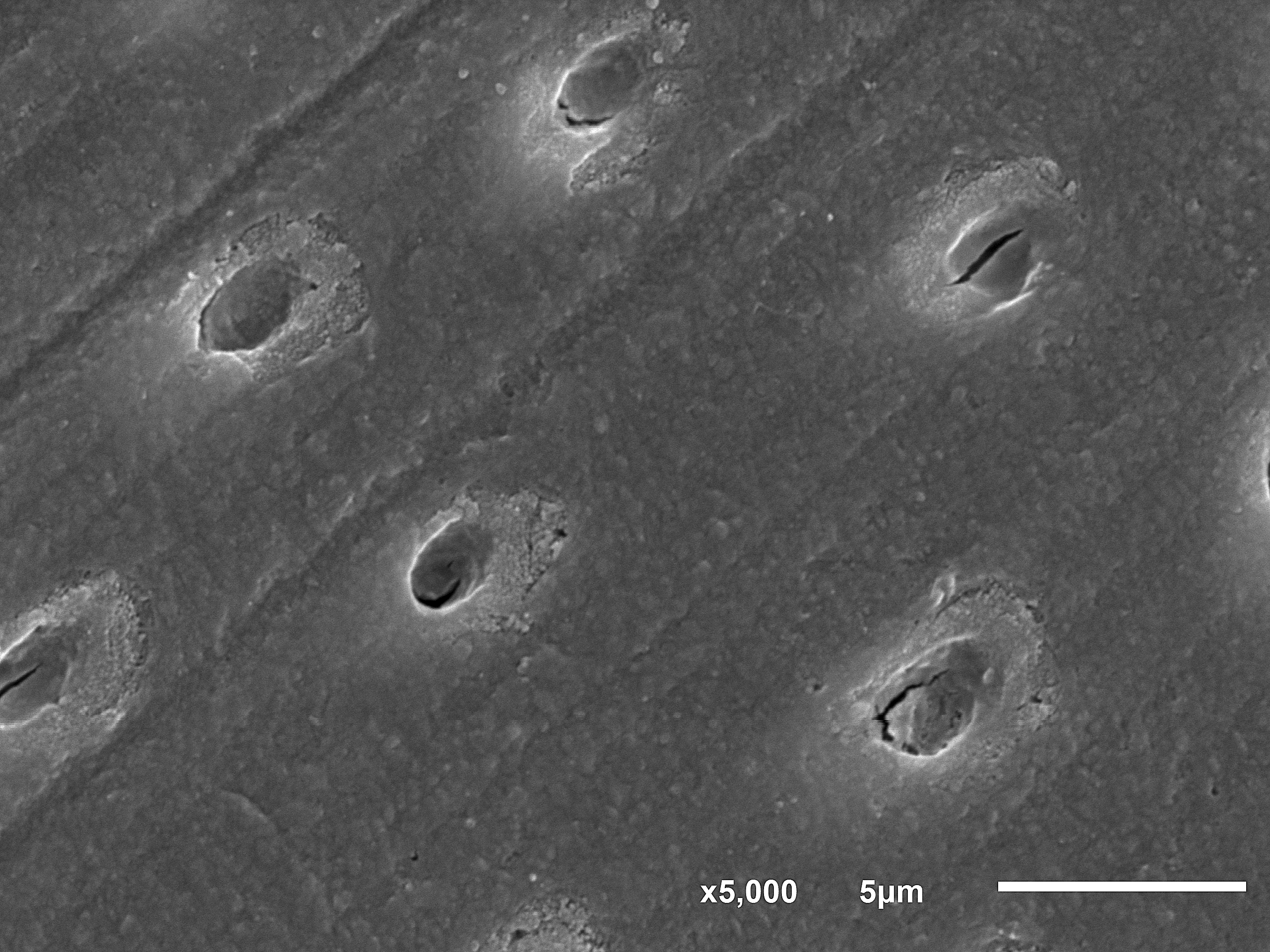

Supplement: S2 Fig — Smear layer was removed by immersion in 0.5% citric acid, natural pH 2.5, for 10 s. Open tubules are clearly visible, the peritubular dentin is preserved. Energy Dispersive X-ray Spectroscopy analysis indicates that there was no relevant demineralisation of the resulting dentine surface (see S1 Table). Original magnification x5000. (TIF) [file pone.0123889.s003.tif]
