# Supplementary material for: Impact of Sn/F Pre-Treatments on the Durability of Protective Coatings against Dentine Erosion/Abrasion
Source: PLoS One. 2015 Jun 15;10(6):e0123889. doi: 10.1371/journal.pone.0123889 (PMC4468142; doi:10.1371/journal.pone.0123889)
Supplement: S1 Table — C, O, P and Ca on dentine surfaces (n = 10 each; wt%, mean±standard deviation) after preparation (with smear layer) and after treatment with 0.5% citric acid (natural pH 2.5) for 10 s (without smear layer). Samples were dried at ambient air, sputter-coated with gold (JFC-1200 fine coater, Tokyo, Japan; 90 s, 40 mA) and investigated at 2000-fold original magnification (JSM-6510, Jeol, Tokyo, Japan equipped with a X-Flash Detector 410-M, Bruker Nano GmbH, Berlin, Germany; acceleration voltage 15 kV, count rates ~1 kcps). Groups sharing the same superscript letter (columns) are not significantly different (t-test for independent samples) (DOC) [file pone.0123889.s004.doc]

|  | C | O | P | Ca |
| --- | --- | --- | --- | --- |
| Without smear layer | 14.9±1.5^a^ | 34.5±1.1^a^ | 14.9±0.9^a^ | 35.8±1.6^a^ |
| With smear layer | 13.4±2.5^a^ | 35.9±2.0^a^ | 15.0±0.8^a^ | 35.7±1.9^a^ |
